# Waardenburg syndrome type I: Dental phenotypes and genetic analysis of an extended family

**DOI:** 10.4317/medoral.20789

**Published:** 2016-03-31

**Authors:** Luciano Sólia-Nasser, Sibele-Nascimento de Aquino, Lívia-Maris-R. Paranaíba, Andreia Gomes, Pedro dos-Santos-Neto, Ricardo-D. Coletta, Aline-Francoise Cardoso, Ana-Cláudia Frota, Hercílio Martelli-Júnior

**Affiliations:** 1 Health Science Program, State University of Montes Claros, Unimontes, Montes Claros, Minas Gerais State, Brazil; 2 Dental School, Federal University of Juiz de Fora, Governador Valadares, Minas Gerais, Brazil; 3 Dental School, State University of Montes Claros, Unimontes, Montes Claros, Minas Gerais State, Brazil; 4 Oral Pathology, School of Dentistry, State University of Campinas, Piracicaba, São Paulo, Brazil; 5 Faculty of Medicine, State University of Montes Claros, Unimontes, Montes Claros, Minas Gerais State, Brazil

## Abstract

**Background:**

The aim of this study was to describe the pattern of inheritance and the clinical features in a large family with Waardenburg syndrome type I (WS1), detailing the dental abnormalities and screening for PAX3 mutations.

**Material and Methods:**

To characterize the pattern of inheritance and clinical features, 29 family members were evaluated by dermatologic, ophthalmologic, otorhinolaryngologic and orofacial examination. Molecular analysis of the PAX3 gene was performed.

**Results:**

The pedigree of the family,including the last four generations, was constructed and revealed non-consanguineous marriages. Out of 29 descendants, 16 family members showed features of WS1, with 9 members showing two major criteria indicative of WS1. Five patients showed white forelock and iris hypopigmentation, and four showed dystopia canthorum and iris hypopigmentation. Two patients had hearing loss. Dental abnormalities were identified in three family members, including dental agenesis, conical teeth and taurodontism. Sequencing analysis failed to identify mutations in the PAX3 gene.

**Conclusions:**

These results confirm that WS1 was transmitted in this family in an autosomal dominant pattern with variable expressivity and high penetrance. The presence of dental manifestations, especially tooth agenesis and conical teeth which resulted in considerable aesthetic impact on affected individuals was a major clinical feature. Clinical relevance: This article reveals the presence of well-defined dental changes associated with WS1 and tries to establish a possible association between these two entities showing a new spectrum of WS1.

**Key words:**Waardenburg syndrome, hearing loss, oral manifestations, mutation.

## Introduction

Waardenburg syndrome type 1 (WS1; MIM #193500) is a rare autosomal dominant disorder characterised by congenital sensorineural hearing loss, dystopia canthorum and depigmentation of the hair (white forelock), skin and iris (heterochromic or hypochromic iris) (1). Musculoskeletal abnormalities of the limbs, Hirschsprung disease and neurological defects may also be observed (2). The prevalence of WS is estimated at 1:42 000, and is responsible for 1-3% of all congenital deafness cases (2). Although first described in northern Europeans (3), the disease has frequently been reported to occur in other ethnic and racial groups (4,5).

Waardenburg syndrome (WS) is classified into four types depending on the additional symptoms present: WS1, WS2, WS3 and WS4. Type I (WS1; MIM #193500) is the classic form of WS with dystopia canthorum (lateral displacement of the inner canthi), whereas type II (WS2; MIM #193510) is characterized by the presence of unpigmented tissue and deafness without dystopia canthorum. Type III (WS3 or Klein-Waardenburg syndrome; MIM #148820) is similar to type 1 WS with additional musculoskeletal abnormalities, and type IV (WS4 or Shah-Waardenburg syndrome or Waardenburg-Hirschsprung disease; MIM #277580) is characterized by the presence of an aganglionic megacolon. A neurological variant of WS4 is a complex neurocristopathy comprised of four distinct syndromes: peripheral dysmyelination neuropathy, central dysmyelination, WS and Hirschsprung disease (PCWH; OMIM 609136), and is a potentially fatal disease (6). The diagnosis of WS1 requires the presence of at least two major criteria, or one major and two minor criteria ( Table 1).

**Table 1:**
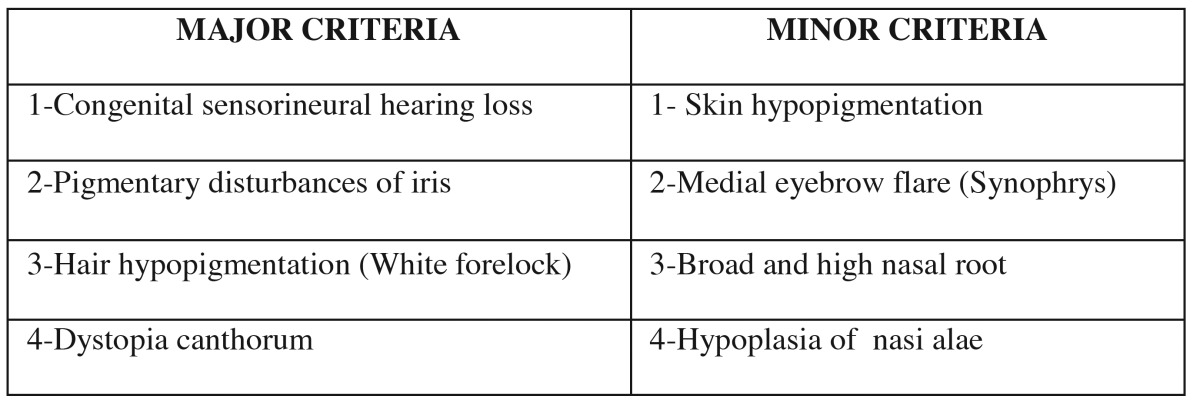
Diagnostic criteria for Waardenburg syndrome types I and II.*

The six genes associated with WS (PAX3, SOX10, MITF, SNAI2, EDN3 and EDNRB) are involved in a network of interactions, and mutations in these genes determine faults in embryogenesis derived from neural crest cells and characterise the phenotypic diversity of WS (1). Mutations in PAX3 (paired box 3 transcription factor) have been found in WS type I and II in numerous studies (7,8).

Oral manifestations of the syndrome have been described in several studies as dental agenesis, cleft lip and/or palate, tooth enamel malformations and fissured tongue (9-13).In this article, a Brazilian family affected by WS1 is reported, and the dental phenotypes, which are very uncommon in this disorder, are detailed. Mutational analysis of the PAX3 gene was also performed.

## Material and Methods

- Patients

**Figure 1:**
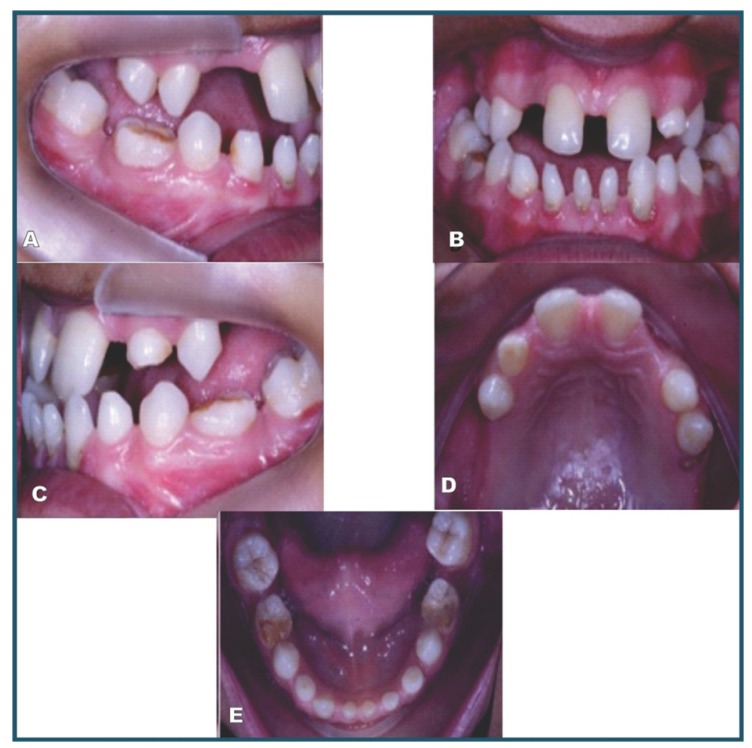
Intraoral manifestations of proband affected by Waardenburg syndrome showing a conoid appearance of 6 teeth and oligodontia of 16 teeth.

**Figure 2:**
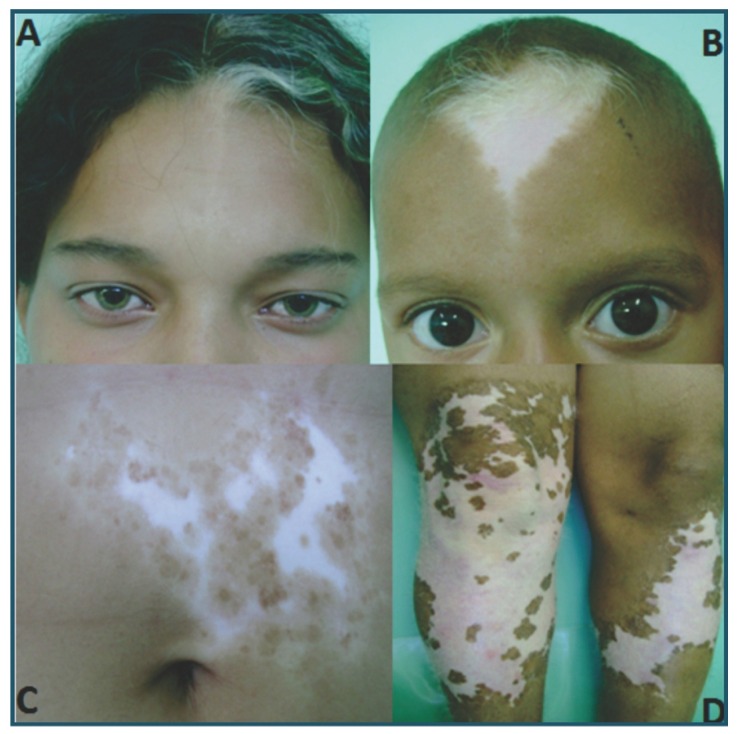
Affected members of family with various clinical associations of the syndrome: white forelock in association with hypopigmented iris and white spots on skin in one of the members of the family, or white forelock and a white spot on the face.

The proband (individual IV-2); a 16-year-old girl, was referred to the Stomatology Clinic of the Dental School at the State University of Montes Claros (Minas Gerais state, Brazil) with a chief complaint of incomplete formation of the teeth. Dental evaluation showed a conical appearance of 6 teeth, oligodontia of 16 teeth and taurodontism in 1 tooth (Fig. 1). Due to the presence of clinical abnormalities such as a white forelock, achromatic spots in the abdominal region and lower limbs, as well as changes in eye colour (Fig. 2), an assessment was requested from the Department of Dermatology and Ophthalmology, which resulted in the diagnosis of WS1.

**Figure 3:**
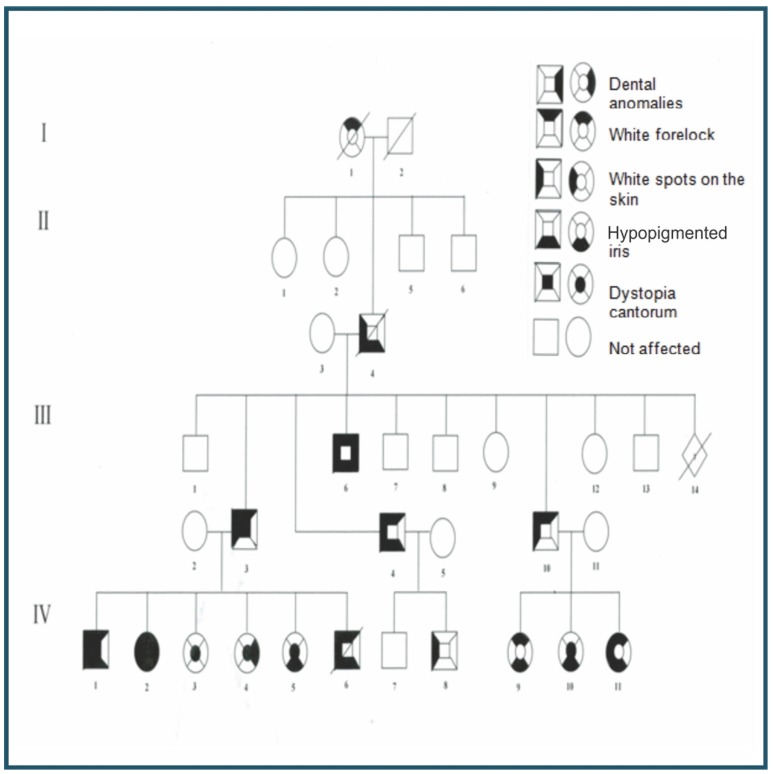
Familial pedigree revealed non-consanguineous marriages and an autosomal dominant pattern of transmission affecting 16 individuals with type 1 Waardenburg syndrome.

A study of the patient’s extended family, which comprised 29 members, was undertaken to determine the inheritance pattern, clinical phenotypes of the disease and expressivity of the syndrome. The family pedigree, including the last four generations, was constructed (Fig. 3). The examination of the eyes involved the measurement of visual acuity, research of dystopia canthorum with a rigid ruler, colour analysis of the iris with a slit lamp and retinal mapping. Imaging analysis, including pictures of the dystopia canthorum, hypopigmentation of the iris and retina (fundus photography), changes in pigmentation of the hair and skin and dental changes was performed. Audiological assessment included otoscopy, as well as tonal and vocal audiometry. The degree of hearing loss was determined according to the method of Lloyd and Kaplan [1978], which classifies hearing loss as follows: 0-25: normal, 26-40: mild, 41-55: moderate, 56-70: moderately severe, 71-90: severe, and above 90: profound hearing loss.

- Dental analysis

**Figure 4:**
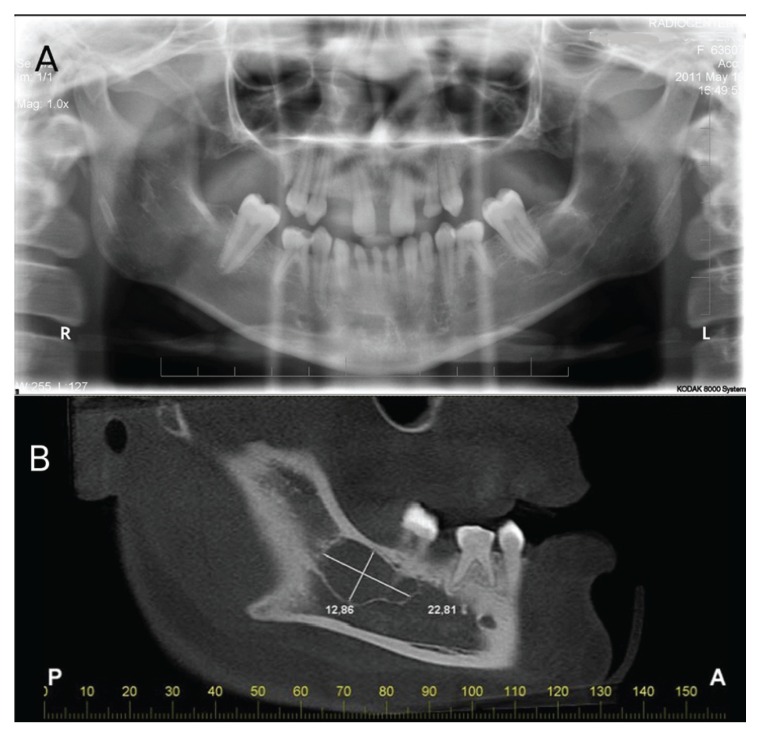
A: Panoramic radiograph of the proband showed dental agenesis, conoid teeth, taurodontism and a radiolucid image of the periapical region of the first inferior molar on the left side. B: Cone beam tomography evidence detailing the radiolucid image of the periapical region of the first inferior molar on the left side, compatible with an idiopathic cavity (simple bone cyst).

Dental evaluation was performed by an experienced dentist who also investigated the presence of environmental factors as related to dental agenesis. Panoramic radiographs and an oral cone-beam computerised tomography scan were performed (Fig. 4). Ethical approval was obtained from the Ethics Committee of the University. Informed consent was obtained from each participant before inclusion in the study.

- DNA extraction and mutational analysis

DNA was extracted from oral mucosa cells as previously described (14). Analysis of exon 2-8 of the PAX3 gene was performed using specific primers to include intron-exon boundaries as previously described (15). PCR products were subjected to bi-directional sequence analysis using the ABI Prism 3500 Genetic Analyser (Applied Biosystems, Foster City, CA, USA).

## Results

Clinical examination of the family members revealed that of the 29 investigated (17 males, 12 females), 16 (55.17%) presented with features of WS1, as described by Read and colleagues [1997] ( Table 1). The gender distribution of the 16 symptomatic patients was 6 males (37.5%) and 10 females (62.5%). The affected patients were aged from 1 to 42 years (mean 17.93 years). Nine patients showed two major criteria (dystopia canthorum and hypopigmentation of the iris, or white forelock and hypopigmentation of the iris) and six members (I-1, II-4, III-10, IV-3, IV-4 and IV-8) showed only one major manifestation, or one major associated with minor manifestations related to WS1 as described in  Table 2.

**Table 2:**
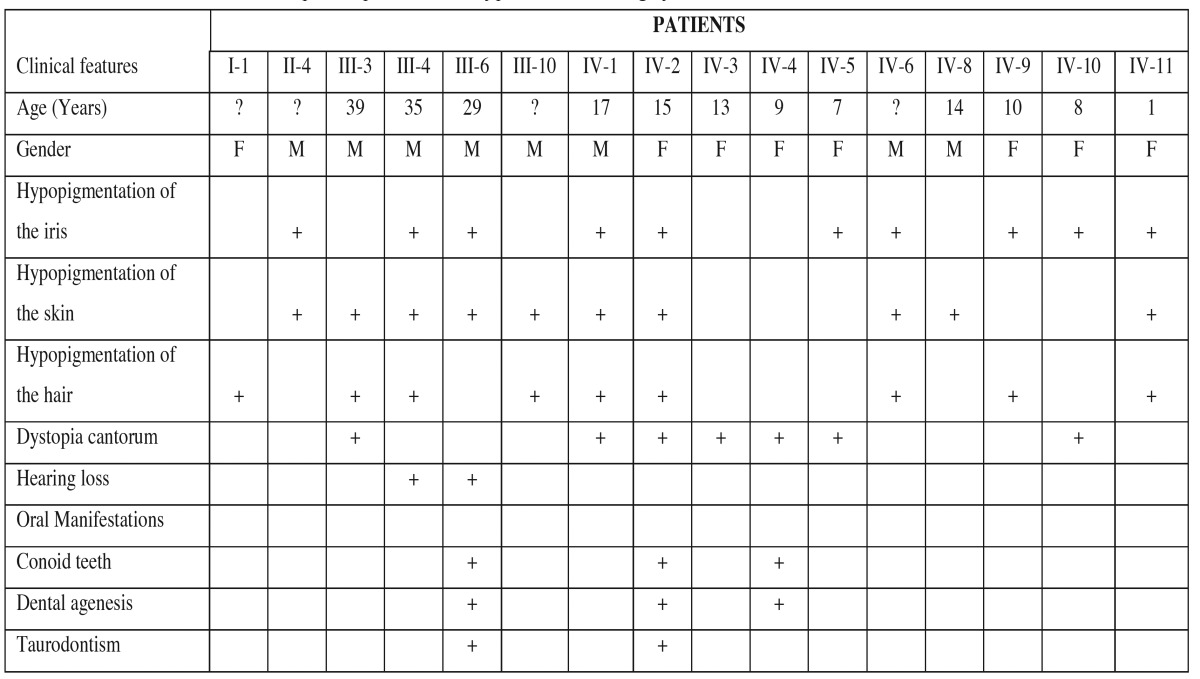
Clinical features of the reported patients with type I Waardenburg syndrome.

There was no history of consanguinity in the family, and it was not possible to examine the earliest generation, although second-generation members stated that their mother (I-1) had a white forelock. All subsequent generations had affected members, with both males and females affected. Segregation of WS1 in this family was consistent with the autosomal dominant mode of inheritance with penetrance appearing to be complete. Clinical analysis demonstrated that the mode of expression varied. Dystopia canthorum (average of 1.96 in affected individuals), which is considered to be the main differential diagnostic criteria for WS1, appears in the third and fourth generations and in seven individuals (III-3, IV-1, IV-2, VI-3, IV-4, IV-5 and IV-10) including the proband (IV-2). The most frequent clinical manifestation was an association of the white forelock, white spots on the skin and a hypopigmented iris (Fig. 2). Two patients had hearing loss (III-3 and III-4).

Dental anomalies such as dental agenesis and conical teeth were identified in three individuals (III-6, IV-2 and IV-4). Patient IV-4 displayed four deciduous conical canines associated with oligodontia of seven teeth. Patient III-6 displayed upper and lower conical canines, and an absence of 18 teeth. The proband (IV-2) displayed a conical appearance in the four lower incisors and two lower canines, and oligodontia of 16 teeth. Additionally, it was observed a radiolucent lesion associated with periapical region of the lower left first molar (Fig. 4). The lesion was submitted to surgical exploration, which did not reveal the presence of liquid or purulent material. The clinical and histopathological aspects were compatible with an idiopathic cavity (simple bone cyst) and was observed bone remodeling in follow up. Taurodontism was identified in two patients, IV-2 (inferior second molar of the right side) and III-6 (first upper and lower molars of the left side) as described in  Table 2. Sequencing analysis failed to identify mutations in the PAX3 gene in the DNA isolated from the members of this family studied.

## Discussion

WS is an inherited disorder characterised by varying degrees of hearing loss and pigmentary anomalies affecting the eye, hair and skin (16). This syndrome is clinically heterogeneous and has been classified into four major types, although 10 subtypes have been reported (17). Specifically, WS1 presents with a typical phenotype called dystopia canthorum (an outward displacement of the inner canthus of the eyes) as well as the main features of WS (1). Here, one family is reported with individuals manifesting features of WS1, in which the transmission occurred as an autosomal dominant trait with a variable phenotype and high penetrance. There was no history of consanguinity in the family to suggest an autosomal recessive inheritance. The variable phenotype identified in this family, including individuals presenting with an isolated feature, is common in WS1 (18). The penetrance of the white forelock, white spots on skin and iris hypopigmentation was very high, but the penetrance of hearing loss was low, and was only observed in 2 (III-3 and III-6) of the 29 family members.

All or most WS type I cases are caused by mutations at the PAX3 locus (8,15). Approximately 70 different PAX3 point mutations have been identified (1). Missense and nonsense mutations, frame shifts, small in-frame insertions or deletions and splice alterations have been described (1). About half of the mutations reported are missense mutations, and half are truncating variations (15). In WS type I, about 95% of PAX3 mutations are located throughout exons 2-6, with the highest rate of mutation found in exon 2 (1). In addition, partial or total gene deletions in PAX3 have been described, and may represent the 10% of cases without identified point mutations (15). PAX3 encodes a transcription factor belonging to the protein-matched (pairedbox) family, and has been implicated in the development of the ear, eye, striated muscles and face (1). PAX3 is also strongly expressed during tooth development (7). Persistent PAX3 expression causes cranioskeletal defects including cleft or shortened palates that result in neonatal lethality (7). In this study, mutations in PAX3 were not found in the DNA isolated from this family. However, genes less commonly associated with WS type I such as SOX10, MITF and SNAI2 were not evaluated.

There are few reports in the literature regarding the oral manifestations of WS. Bandyopadhyay and co-workers (1999) identified only dental agenesis of the lower lateral incisors in patients affected by this disorder (11). Cleft lip and/or palate have also been described in WS (19,20) and undefined jaw malformations and mandibular prognathism were described as possibly being related to WS (10-12). Irregular teeth, abnormalities of the tooth enamel (not specified), a high palate and a fissured tongue also have been included as part of the clinical spectrum of the oral manifestations of patients affected by the syndrome (21,22). In this study, a number of oral alterations such as dental agenesis, taurodontism and conical teeth were found to be related to WS. The simple bone cyst observed was not considered as characteristic of the syndrome, as it is found in the general population, and has not been described in WS previously and has a possible traumatic etiology.

Agenesis of one or several teeth (oligodontia) is the most common disorder in human tooth development. Dental agenesis has been noted as a multifactorial condition with genetic, environmental, pathological and evolutionary influences (23). No environmental factors were identified in the present study as being related to dental agenesis, and despite the small number of individuals in the affected family, autosomal dominant inheritance with incomplete penetrance and variable expressivity can be identified. The prevalence of tooth agenesis varies according to the class of the tooth, and permanent dentition has been shown to be more affected than deciduous dentition (23). The prevalence of multiple dental agenesis (the absence of four or more teeth, other than the third molars) is estimated to be approximately 0.25% of the population (24). Studies involving the Caucasian population have shown that the second premolar and lateral incisor are the most commonly missing teeth (25,26). In this study, the majority of missing teeth were upper lateral incisors (12.19%) followed by lower lateral incisors, lower central incisors, upper and lower second premolars, second molars and third molars, each with a frequency of 9.75%. It was also observed that agenesis of the maxillary lateral incisors surpassed that of the third molars in this family. Epidemiologic studies have shown that dental agenesis can manifestal one or as part of a syndrome (27,28). There are more than 60 syndromes associated with dental anomalies categorised in Online Mendelian Inheritance in Man (OMIM), implying that common molecular mechanisms may be responsible for changes in teeth and other organs (http://ncbi.nlm.nih.gov/OMIM). In this study, the presence of multiple tooth agenesis was observed in three members of the family exhibiting WS1; one member of the third generation and two of the fourth.

Conical teeth have a prevalence of approximately 1% in the general population (29). The upper lateral incisors form the class of teeth that is most affected by this change, and this is most common in females (30). In this study, the lower canine teeth were most frequently conoid (42.85%), followed by the lower incisors and canines, both with a frequency of 28.58%, characteristics that contradict the main studies on conical teeth. Females prevailed with a 2:1 ratio. Conical teeth are often associated with agenesis, and also have an autosomal dominant pattern of inheritance (30). They are often found associated with genetic disorders such as the ectodermal dysplasias, Rieger syndrome, incontinence, onicodermal and dento-pigmented syndrome (28). Phenotypic heterogeneity has been significant among families showing variable expression, and this is characteristic of WS. The presence of strongly expressed dental alterations, especially conical teeth and tooth agenesis, results in considerable aesthetic impact on affected individuals, and this requires a multidisciplinary approach for all family members. Moreover, early diagnosis of these alterations can help to improve the quality of life of affected patients.

## Conclusions

The results presented here confirm that WS1 was transmitted in this family via an autosomal dominant pattern with variable expressivity and high penetrance. The presence of mutations in the PAX3 gene was not detected in this family. It was not possible to establish a clear association between dental alterations and WS1 in the present study. Research involving larger samples, with a focus on other genes associated with WS, is required to improve the understanding of the mechanisms involved in the clinical manifestations of this rare syndrome. This will allow medical professionals to offer patients a careful genetic counselling regime and a more complete treatment.

## References

[1] Pingault V, Ente D, Dastot-Le Moal F, Goossens M, Marlin S, Bondurand N (2010). Review and update of mutations causing Waardenburgsyndrome. Hum Mutat.

[2] Read AP, Newton VE (1997). Waardenburg syndrome. J Med Genet.

[3] Waardenburg PJ (1951). A new syndrome combining developmental anomalies of the eyelids, eyebrows and nose root with pigmentary defects of the iris and head hair and with congenital deafness. Am J Hum Genet.

[4] Otman5 SG, Abdelhamid NI (2005). Waardenburg syndrome type 2 in an African patient. Indian J Dermatol Venereol Leprol.

[5] Morin M, Vinuela A, Rivera T, Villamar M, Moreno-Pelayo MA, Moreno F (2008). A de novo missense mutation in the gene encoding the SOX10 transcription factor in a Spanish sporadic case of Waardenburg syndrome type IV. Am J Med Genet.

[6] Inoue K, Khajavi M, Ohyama T, Hirabayashi S, Wilson J, Reggin JD (2004). Molecular mechanism for distinct neurological phenotypes conveyed by allelic truncating mutations. Nat Genet.

[7] Wu M, Li J, Engleka KA, Zhou B, Lu MM, Plotkin JB (2008). Persistent expression of Pax3 in the neural crest causes cleft palate and defective osteogenesis in mice. J Clin Invest.

[8] Chen H, Jiang L, Xie Z, Mei L, He C, Hu Z (2010). Novel mutations of PAX3, MITF, and SOX10 genes in Chinese patients with type I or type II Waardenburg syndrome. Biochem Biophys Res Commun.

[9] Silan F, Demirci L, Egeli A, Egeli E, Onder HI, Ozturk O (2004). Syndromic etiology in children at schools for the deaf in Turkey. Int J Pediatr Otorhinolaryngol.

[10] Fisch L (1959). Deafness as part of a hereditary syndrome. J Laryngol Otol.

[11] Bandyopadhyay S, Prasad S, Singhania PK (1999). Partial anodontia in a case of Waardenburg's syndrome. J Laryngol Otol.

[12] Sommer A, Bartholomew DW (2003). Craniofacial-deafness-hand syndrome revisited. Am J Med Genet A.

[13] Salvatore S, Carnevale C, Infussi R, Arrico L, Mafrici M, Plateroti AM (2012). [Waardenburg Syndrome: a review of literature and case reports]. Clin Ter.

[14] Aidar M, Line SR (2007). A simple and cost-effective protocol for DNA isolation from buccal epithelial cells. Braz Dent J.

[15] Pandya A, Xia X, Landa B (1996). Phenotypic variation in Waardenburg syndrome: mutational heterogeneity, modifier genes or polygenic background?. Hum Mol Genet.

[16] Newton VE (2002). Clinical features of the Waardenburg syndromes. Adv Otorhinolaryngol.

[17] Wang J, Li S, Xiao X, Wang P, Guo X, Zhang Q (2010). PAX3 mutations and clinical characteristics in Chinese patients with Waardenburg syndrome type 1. Mol Vis.

[18] Arias S, Mota M (1978). Apparent non-penetrance for dystopia in Waardenburg syndrome type I, with some hints on the diagnosis of dystopia canthorum. J Genet Hum.

[19] Tay CH (1971). Waardenburg's syndrome and familial periodic paralysis. Postgrad Med J.

[20] Asher JH Jr, Friedman TB (1990). Mouse and hamster mutants as models for Waardenburg syndromes in humans. J Med Genet.

[21] Bondurand N, Dastot-Le Moal F, Stanchina L, Collot N, Baral V, Marlin S (2007). Deletions at the SOX10 gene locus cause Waardenburg syndrome types 2 and 4. Am J Hum Genet.

[22] Dourmishev AL, Dourmishev LA, Schwartz RA, Janniger CK (1999). Waardenburg syndrome with facial palsy and lingua plicata: is that a new type of disease. Cutis.

[23] Vastardis H (2000). The genetics of human tooth agenesis: new discoveries for understanding dental anomalies. Am J Orthod Dentofacial Orthop.

[24] Sarnäs KV, Rune B (1983). The facial profile in advanced hypodontia: a mixed longitudinal study of 141 children. Eur J Orthod.

[25] Larmour CJ, Mossey PA, Thind BS, Forgie AH, Stirrups DR (2005). Hypodontia-a retrospective review of prevalence and etiology. Part I. Quintessence Int.

[26] Dechkunakorn S, Chaiwat J, Sawaengkit P (1990). Congenital absence and loss of teeth in an orthodontic patient group. J Dent Assoc Thai.

[27] Jorgenson RJ (1980). Clinician's view of hypodontia. J Am Dent Assoc.

[28] Li X, Venugopalan SR, Cao H, Pinho FO, Paine ML, Snead ML (2014). A model of molecular underpinnings of tooth defects in Axenfel-Rieger syndrome. Hum Mol Genet.

[29] Bäckman B, Wahlin YB (2001). Variations in number and morphology of permanent teeth in 7-year-old Swedish children. Int J Paediatr Dent.

[30] Alvesalo L, Portin P (1969). The inheritance of missing, peg-shaped and strongly mesiodistally reduced upper lateral incisors. Acta Odontol Scand.

